# Educating undergraduate occupational therapy and physiotherapy students in motivational interviewing: the student perspective

**DOI:** 10.1186/s12909-019-1560-8

**Published:** 2019-04-27

**Authors:** Meriel Norris, Gail Eva, Jennifer Fortune, Tai Frater, Jeff Breckon

**Affiliations:** 1 Department of Clinical Sciences, Brunel University London, Uxbridge, UB8 3PH UK; 2 Faculty of Health and Life Sciences, Oxford Brookes University, Marston Road Campus, Oxford, OX3 0FL UK; 3 Academic Unit of Neurology, Biomedical Sciences Institute, Trinity College Dublin, Dublin, Ireland; 4 Advanced Wellbeing Research Centre, Sheffield Hallam University, Collegiate Crescent, Sheffield, S10 2BP UK; 5 College of Health and Life Sciences, Brunel University London, Kingston Lane, Uxbridge, UB8 3PH UK

**Keywords:** Motivational interviewing, Physiotherapy, Occupational therapy, Education, Students, Qualitative

## Abstract

**Background:**

Motivational Interviewing (MI) is an evidenced based talking therapy designed to affect client Health Behaviour Change. Previous research indicates that Allied Health Professionals (AHP) can effectively use the approach and training at pre-registration level has been piloted. However, student experiences of training is underexplored.

**Aim:**

To explore Physiotherapy and Occupational Therapy students’ experiences of training in and implementation of Motivational Interviewing.

**Methods:**

Four focus groups including 24 undergraduates (14 OT and 10 PT) were conducted at the completion of the training and a subsequent clinical placement. Transcribed texts were analysed thematically. Data were triangulated with student written post-it notes and open questions in a post training questionnaire.

**Results:**

Two overarching themes were developed from the data. *Learning different ways to interact and the challenge of transformation* illuminates specific aspects of the training which enabled learning as well as areas of contention. *Using the spirit of MI, but not every contact counts* highlights the facilitators and challenges of implementation on placements.

**Conclusions:**

Motivational interviewing is a useful addition to training neophyte health students. Key skills were adopted and in some cases transferred into practice. The process of learning indicates areas of potential improvement to enhance relevance of practice scenarios. The transfer to practice is more complex illustrating a need to negotiate professional and institutional expectations which should be considered in training.

## Background

Motivational Interviewing (MI) is a collaborative, goal orientated communication approach designed to address ambivalence in behaviour change and empower the individual to take responsibility for their own health [1]. Originating in substance dependency treatment [2] MI has emerged as an effective approach to promote behaviour modification and chronic disease management [3–5] and consequently has been adopted across a broad spectrum of health care professions to support client behaviour change and self-management [6, 7].

As the empirical evidence to support the effectiveness of MI has grown it has been increasingly incorporated into undergraduate and graduate healthcare curriculums [8, 9]. MI training has been successfully implemented in a range of higher education disciplines including medicine [10–13], pharmacy [14], dentistry [15, 16] and allied healthcare [17, 18] with demonstrated positive effects on MI knowledge, confidence and skill performance.

Despite the demonstrated benefits of MI training to student competence little research to date has examined student perception of MI training. While evaluation studies have shown that MI training is typically perceived as valuable, relevant to practice and feasible to integrate [18–20] many unanswered questions remain regarding trainee preference and the keys to effective training and implementation. Research concerning student experience of integrating MI into their professional practice based on training is even sparser. Although the limited available evidence demonstrates positive effects on patient outcomes in clinical practice subsequent to MI training in student cohorts [16], longitudinal evaluation of skill retention following transition from training to clinical practice are uncommon in the literature [13, 17, 21]. Consequently, there is a dearth of research regarding student perspectives on the experience of implementation and transferability of MI skills. Prior studies examining the application of MI among clinicians have indicated the potential utility but also highlighted the implementation challenges including limited time and patient resistance [22] suggesting a need to focus on the quality of implementation not just its training [19, 23]. Further exploration of the student specific challenges facing implementation to practice is warranted.

Given the growing use of MI it is of critical importance to review and evaluate the experiences of students trained in it to optimise the educational benefits of training and ensure effective application of MI skills in clinical practice. The aim of this paper is to explore student experience of MI training and implementation within an allied health professional context. The study reported in this paper is part of a larger training programme designed to pilot an interdisciplinary approach to learning MI for pre-registration OT and PT students [17]. The intervention components, described in detail elsewhere, consisted of an introductory lecture, followed by three days of experiential training on both the relation (or spirit; evocation, empathy, collaboration, compassion) and technical components (OARS; open questions, affirmations, reflective listening and summaries) of MI, and was facilitated by a member of the Motivational Interviewing Network of Trainers (MINT). Assessments of competence were applied at baseline; immediately post training; and following the subsequent clinical placement. The training was delivered as an additional and optional supplement to core curricula activities which students volunteered to undertake.

## Methods

A qualitative methodology drawing on interpretative traditions was selected as the most appropriate approach to explore the students’ experiences of the MI training and use on placement [24].

Data were collected through focus groups which enabled exploration of individual experiences alongside the additional insights gleaned from the group interaction, enhancing depth of insight and narrative development. Previous authors have suggested that this approach has particular salience for previously formed groups [25], such as the training groups in this study.

All students who participated in the training programme were invited to join one of four focus groups. Written informed consent was confirmed prior to inclusion. All students undertook a 6 (PT) or 8 (OT) week clinical placement after they completed the MI training programme and the focus groups were held immediately following the end of that placement. Due to course timetables these were different for OT and PT students and as a result the focus groups were profession specific.

Each focus group was led by a facilitator (MN or GE) from the same profession as the students, who had also undertaken MI training as part of the study. They therefore had shared experience of both the training and the clinical context in which the students were applying their new knowledge. All focus groups were audio-recorded, but a co-facilitator (MN or GE) also acted as scribe to summarise thoughts on flip charts. This acted as a form of member checking as participants were asked to review the notes during the discussions. While the shared experience between student and facilitator could be perceived as a potential influence on the data production, it has also been argued that shared experience enhances both the willingness to share information and the relevance of the facilitation [24].

A topic guide was developed and discussed by the research team prior to confirmation. Indicative content included: reflections on motivation to undertake training; a post-it note activity focusing on their learning experience in which students individually identified key learning points and features which were then organised and discussed by the group; reflections on use of MI in clinical placements; and relevance of such training on an undergraduate course.

### Analysis

The recordings were transcribed verbatim and an adapted thematic analysis [26] was conducted following several stages.

- Stage 1: the transcribed texts of the profession-specific focus groups were analysed for content by the facilitator of that focus group. This involved close reading and re-reading of the texts followed by line and group coding of content. The individual post-it notes used in the focus groups were checked to ensure all content was included in the group codes.
- Stage 2: the two focus group/analysis leads compared and discussed the resultant group codes. Similarities and differences were discussed and an overarching thematic map was agreed.
- Stage 3: the major themes from the focus groups were discussed with the wider research team in order to enhance depth in analysis.
- Stage 4: Comparison with other data sources from the study e.g. an open question from a questionnaire completed immediately post training acted as a form of triangulation of methods to ensure that all responses were appropriately captured.
- Stage 5: the final themes and sub-themes were confirmed and indicative quotes added to enhance transparency.

Rigour of the analysis was enhanced during stages two to four through critical dialogue and a level of triangulation [27, 28].

## Results

Twenty five undergraduate (15 OT and 10 PT) students undertook the MI training. All but one OT student, who was on an extended placement, participated in the focus groups. The focus groups lasted between 67 and 72 min (mean 68.75 min). Participants were aged 19–46 years (mean = 25.7, SD = 7.2). Eighty-eight percent of participants (*n* = 22) were female. Participants had completed 1–6 years in higher education (mean = 2.9, SD = 1).

Two themes developed from the data specifically relate to the aims of this paper. Learning different ways to interact and the challenge of transformation illuminates specific aspects of the training which enabled learning, as well as areas of contention. Using the spirit of MI, but not every conversation counts highlights the facilitators and challenges of using MI on clinical placements.

### Learning different ways to interact and the challenge of transformation

The experiential nature of the training was identified as a key benefit by the students. It allowed them to reflect on their current communication habits and provided them with an opportunity to observe, experience and importantly practice new ways of comunicating. Continual performance of MI was integral to the process of learning as well as a product of training.

MI was modelled by the trainer in all interactions with students, both though explicit use of the tools, and implicitly, by collaborating with the students in learning. Teacher/student hierarchies were diminished in the same way an MI practitioner would with a patient.

*Yeah, it’s like breaking down that I’m here to teach you, you’re going to take it in, that’s the end of it but it’s more like okay, I’m actually here to guide you to get this. It makes it more approachable as well.* (OT FG2)

In addition to motivating the students and making the process of learning more accessible, this modelling had two distinct effects on the training. The first was that the students gained an understanding of how future MI could be implemented. Observing an expert fluently embed MI into the entire teaching process inspired the students to imagine a future skilful self while they were currently grappling with the basics of MI practice.

*“It reassured me personally because when you obviously start a skill you start like actively thinking, oh am I making a reflection now, oh am I summarising now, that kind of thing. But him [the trainer] just doing it fluently gave me essentially the hope that it will come more fluidly and I won’t actively have to think about it, but it will be more of a conversation rather than like a kind of an active effort on my path to try and get something.” (PT FG1)*

As the training evolved this extended to a collective educational experience as students learned from each-others attempts and input, building their confidence through collaborative practice.

The second result of the modelling was that students experienced MI themselves, which they found invaluable. Although students were consciously aware that the conversations with the trainer were part of an educational exercise they nonetheless found themselves reflecting on their personal behaviour. This first-hand experience illuminated the power of MI and had a strong impact on their motivation to engage in the training.

*Like it can work on you, it can work on anyone so that also helped to show me that it is effective because if... I was starting to kind of rethink things, like oh gosh yes like why did I do that, or why haven’t I done that, then it would be effective on other people as well.* (PT FG1)

While vicarious learning and experience was deemed important and inspiring, skills practice was an essential component of the training and was highly valued by the students. Students were given role play scenarios of the kind typically encountered by health care staff in behaviour change conversations, such as smoking cessation, weight loss, and increasing physical activity. Patients in these vignettes were typically older, juggling complex life demands. These enabled students to receive immediate feedback which helped to hone their skills and were also deemed to enhance their empathy through greater understanding of complex situations.

Nevertheless, they felt somewhat artificial, being far removed from students’ own experiences.

*I think because we’re all sort of young adults, we don’t really have any of the complications, like a lot of us aren’t diabetic, all that kind of things, so we wouldn’t have any exposure to how someone would be feeling like that.* (PT FG2)

For this reason, ‘real-play’ was also used, where students practiced MI in patient-therapist pairs, drawing on real areas of ambivalence in their own lives. Students reflected that real-play resulted in more natural conversations, grounded in the complexities of every-day life and as a consequence more opportunities for the skills of MI to be utilised. It was considered more hard-hitting and relevant then role-play.

This was highly valued, but also posed some challenges, for example, choosing a ‘just right’ topic to discuss. Something too trivial rendered the exchange too superficial to be meaningful, but more personally challenging topics ran the risk of disomforting disclosure which had to managed appropriately.

*I found the real plays incredibly interesting and I was happy to throw open the closet and listen to other people throw open their closet. There was safety in knowing that nobody was going to go out and gossip and I thought it was a really interesting experience.* (OT FG2)

While this student was comforted by the ‘ground rules’ of engagement, for others, who you were talking to became important, with some reluctance to share with strangers in the group.

A further issue was the relevance of the specific topics in role or real play. Students emphasised that to enhance their skill acquisition and application, scenarios needed to be directly transferable to their encounters with clients in their practice placements.. Future suggestions were scenarios where patients demonstrated ambivalence towards engaging in rehabilitation activities and taking responsibility for activities outside of sessions.

Despite these challenges in approachthe students recognised changes in their communication as a result of the training. They described how they found themselves listening more, stepping back from the expert role and applying the MI techniques of open questions, affirmations, reflective listening and summarising.

### Using the spirit of MI, but not ‘every conversation counts’

In general, the students found that MI enhanced their engagement with patients during their practice placement, as they had learned to listen more (and more effectively) and speak less.

*I learned a lot, by truly stopping and giving time to understand where an individual is coming from, giving them the space to explore their own personal barriers. (OT FG1)*

*It sort of opened my eyes to more asking patients why. So on my first placement, if a patient declined doing something I would be like, okay so you don’t want to do it. Whereas now I’m more interested to say why? (PT FG2)*

Some were able to give detailed descriptions of how they had used MI to achieve rehabilitation goals with certain individuals. These varied from enhancing commitment to inpatient rehabilitation, to exploring longer term physical activity options, and incorporated a range of MI skills.

Many more described using specific MI techniques (especially reflections and open questions) in therapy interactions, indicating that perhaps some MI components were more helpful or accessible to novice practitioners.

*On placement I only really used open questions and reflections, because that helped me guide a conversation…I was doing it more to actually get more information out, so I was just sort of like used the principles to help me in my situation (Physio FG2)*

Nevertheless, a number of barriers were identified that restricted their ability to use MI in practice. Students felt that their MI training assumed that patients perceived the need for a health behaviour change (at some level), had an interest in achieving that change (although possibly resistant), and required help. The reality on practice placements was different, and students questioned the idea that every conversation could be made to count. They observed patients typically falling into different categories, for example those able to change without help, those whose expectation that therapists would fix the problem hindered their engagement, those too ill to engage or lacking cognitive capacity, and those with life-changing illness who were unable to engage with a difficult and uncertain future. In these circumstances, they felt unable to use MI skills.

*I found on placement … either people were ready to change and they were doing that actively themselves or people were not going to change at all, and therefore I didn’t really have a chance to use my motivational interviewing that much* (Physio FG2)

*Mine was in neuro rehab so we were dealing with inpatients whose lives changed dramatically who probably weren’t going back to the same life they had anyway so talking about their lifestyle changes and motivation to do things was just not appropriate.* (OT FG2)

When confronted with patients who were not typically ambivalent (as presented in the training), students struggled to see the relevance of the approach. Interestingly, they attributed the difficulty to a patient/MI mismatch, rather than their own inexperience.

Student’s also experienced organisational barriers to using MI on placement, for example very brief contact time with patients, and roles that were very rigidly defined.

*My placement was in orthopaedics and […] sometimes patients are discharged on day two, and you barely even see them. Even if you do see them, it’s only for fifteen minutes assessing mobility and transfers and then they’re discharged.* (OT FG2)

The patient, in these circumstances, was framed within an institutional to-do list where engaging with their aspirations and needs was given little importance or space.

A further barrier was their practice educators’ awareness of MI. Students had difficulty getting advice on the appropriate use of MI in specific circumstances, or were explicitly directed to take an expert role despite indications that a collaborative approach would have been appropriate as the following extended quote demonstrates.

*There was a lady who didn’t want to be discharged until she was like completely able to go home and [my educator] was just pushing rehab, pushing and pushing: “Go ask her about rehab.” “Oh I have already asked.” “Go ask again because she has to go to rehab.” You could tell the lady just didn’t want hear it. … It is quite hard to try and implement it when your educator doesn’t really know about it and to them it might seem like you are wasting time.* (Physio FG1)

Students perceived that educators wanted them to focus on rehabilitation, with general health issues such as weight management or smoking cessation seen as the remit of dietitians, nurses and other members of the multi-disciplinary team.

Taken together this theme demonstrates the potential that the short training has to impact on practice placements. Nevertheless, these narratives also highlight very practical challenges that faced students on placement which may impact on their capacity to incorporate approaches such as MI.

## Discussion

This examination of neophyte OT and PT student experiences of pre-registration training in MI suggests that it has promise as a communication approach, which students perceive enhances their skills as a professional, and can be applied in some contexts of practice. In addition, this study highlights a number of key points which can inform consideration of wider implementation in AHP training.

First is the importance of practice within the training. This emphasis was both clear in this study and supported by other related papers [18, 23, 29]. For Schoo and colleagues [18], the relevance of this practice was enhanced through the addition of a reflective piece and that could be considered.

Critical here was the utility of the practice scenarios. Role-play, while valuable in enhancing empathy, was deemed somewhat distant from the students’ experience and therefore a challenge to engagement. In an attempt to ameliorate these barriers real-play was utilised but this lacked specificity to the clinical environment. As a consequence, the simulations used through both role and real play did not reflect clinical conversations for therapists and therefore were difficult to transition to practice. It was evident that efforts need to be made to create scenarios more relevant to the students, either personally or to their practice, to facilitate development and that transition. The need for skill specific examples has been noted previously [19]. Given the paucity of educational literature on MI with AHPs and its still limited use in practice, development of such resources is work to be done.

A second point noted was the experiential nature of the training. In line with existing research [6, 8, 29] guided experiential practice and opportunities to directly apply skills were highly valued by participants. These experiences, facilitated by a skilled trainer through observation, standardised patient exercises and role play promoted understanding of the MI process and enhanced student engagement. The importance of the skilled trainer for both demonstrating MI in practice but also facilitating the students to experience MI personally was evident. Literature from other forms of simulation also note the importance of such experience [19]. The implications of this for delivery of training are clear in relation to trainer expertise, but also maintaining a trainer student ratio that allows for that personal interaction.

Third, the findings related to the transfer to practice are particularly important given the noted dearth in this area. The evidence that a number of the students tried to implement MI while on placement is positive. Previous studies noted that students had not successfully negotiated this transfer, in part related to a lack of confidence in their ability [23]. This was not explicitly identified in this study and indeed opportunities to reflect on individual competence and confidence were notably absent. Interestingly Schoo and colleagues [18], found that the OT and PT students over-rated their skill level post training. While this was not assessed in this study, it may suggest that consideration of perceived confidence in specific skills is worth further exploration to enhance appropriate self-awareness.

Within this study and across other related research, there was a strong sense that on clinical placement students related to the spirit of MI and specific skills (OARS) which they had drawn from their training, rather than taken directly from the training scenarios. Something similar is noted even in simulated environments [18]. The students in this study linked this in part to the lack of specificity of scenarios discussed above but also the practicalities of transfer into the clinical environment. Barriers such as time, a focus on patient education as an expert, tensions when other professionals do not use it and the underpinning philosophy of the setting have been identified in previous research [19, 29] and are supported in this study. These suggest a need for further training in the wider clinical environment, potential adjustments to training to explicitly discuss these areas and ways to manage them in practice, but also increase dialogue between University-based education and that within the clinical environment. If enhanced communication skills such as MI are to be effectively incorporated into pre-registration training with an expectation of development while on placement, then this may need to be negotiated with clinical educators. Furthermore, consideration in future could also be given to explicit documentation within assessment criteria where and if appropriate. However, adjusting learning outcomes of placements to incorporate the use of MI assumes that the placement has capacity to deliver on this both in relation to client case-load and supervisor expertise, neither of which were fully supported by this study.

Finally, while the literature base is small, there is a general sense that students perceive that MI is a valuable tool that should be incorporated in pre-registration training as it has the potential to improve patient care [19, 20, 23, 29]. This study adds to that call and gives explicit focus on how students feel that could be done more effectively.

## Limitations

This was a small study based in in one Higher Education Institution in the South East of the UK. The students volunteered for this training and the potential influence of motivation is noted. While the training was undertaken by external personnel, the involvement of staff members may have influenced the students because of perceived institutional investment. Nevertheless, the range of responses would challenge that concern.

## Conclusion and practice implications

MI was seen as a useful addition to pre-registration therapy students. Key skills were adopted and in some cases effectively transferred into practice. The study highlights facilitators and barriers to effective learning and hidden curricula effects that hinder skills transferability to clinical placement. Suggestions to address these include consideration of skill level of facilitator, specificity of scenarios, closer links between educators and those in practice and understanding and negotiation of institutional demands.
